# The Role of Smallholder Pig Farmers in the Biosecurity of Pig Diseases in the Eastern Cape Province of South Africa

**DOI:** 10.1155/vmi/4755096

**Published:** 2025-05-26

**Authors:** Vincent Simbizi, Rebone Moerane, Bruce Gummow

**Affiliations:** ^1^Discipline of Veterinary Science, James Cook University, Townsville, Queensland, Australia; ^2^Department of Production Animal Studies, Faculty of Veterinary Science, University of Pretoria, Onderstepoort, Gauteng, South Africa

**Keywords:** antimicrobial resistance, biosecurity, farming practices, pig diseases, remedies, smallholder pig farmers

## Abstract

Biosecurity forms an important component of preventing disease transmission. However, data on the demographics and practices of smallholder pig farmers in Southern Africa are scant, and little is published on the biosecurity related to these farms. A questionnaire survey was, therefore, carried out in the Eastern Cape Province of South Africa to describe the demographics and practices of smallholder pig farmers and to understand their role in the biosecurity and prevention of pig diseases. Females represented 52% of pig farmers and reflect the cultural importance of pig farming in Xhosa culture. All the farmers who were interviewed had poor biosecurity measures on their farms. A low level of education, lack of training and reliance on remedies to treat and prevent pig diseases were key findings for the majority of farmers. Farmers had a poor knowledge of correct antibiotic use, which could contribute to antimicrobial resistance (AMR). Smallholder farms were found to frequently involve free-ranging pigs, swill feeding and informal trading, practices known to contribute to the spread of communicable pig diseases such as foot and mouth disease and African swine fever. Smallholder pig farms are, therefore, a potential risk for disease incursion and spread of communicable diseases within a region. Cost-effective biosecurity measures and marketing opportunities will help to prevent pig diseases, while a continuing education programme will modernise the rural pig industry and reduce the impact of AMR.

## 1. Introduction

The increasing human population in Southern African countries has put pressure on all stakeholders to improve income generation and food security. Due to the low capital investment needed for informal pig keeping, there has been a steady increase in the number of smallholder pig farms [1–3].

Biosecurity measures for smallholder pig farms in the Eastern Cape Province (ECP) of South Africa and in many sub-Saharan African countries remain a challenge. In the absence of vaccines for some pig diseases such as African swine fever (ASF) or their inaccessibility by resource-poor farmers, improved biosecurity is still the only way to achieve disease prevention and to control outbreaks [4]. This was seen during the last outbreak of classical swine fever (CSF) in the province where losses could have been prevented by applying better biosecurity measures [5]. Biosecurity measures applicable to smallholder pig farmers should be risk-based, accepted by farmers, feasible and cost-effective [4]. In the context of this paper, we refer to biosecurity at a farm level.

There are few studies on communicable pig diseases in smallholder communities of ECP and those that have been published only focus on a limited number of districts and provide little information on biosecurity of smallholder pig farms in the province [6–8].

Similarly, limited studies on the demographics and practices of smallholder pig farmers in the ECP have been conducted [9–11], and there is currently no active surveillance for pig diseases in rural domestic pigs. Hence, little is known about disease transmission and biosecurity within the rural pig farming sector of the ECP. The objectives of this study were, therefore, to use a questionnaire survey to describe the demographics and practices of smallholder pig farmers in the province to understand their role in biosecurity and prevention of pig diseases.

## 2. Materials and Methods

### 2.1. Study Design

#### 2.1.1. General Overview

The study comprised an interview-based questionnaire survey targeting smallholder pig farmers in the ECP conducted from February to June 2019.

#### 2.1.2. Study Area

**Figure 1:**
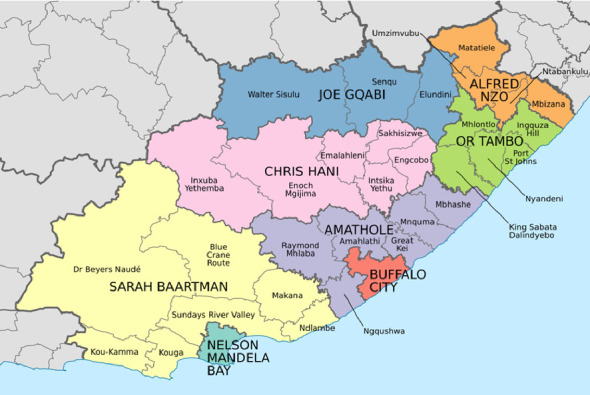
A map of ECP showing its districts and municipalities (https://commons.wikimedia.org/wiki/File:Map_of_the_Eastern_Cape_with_municipalities_named_and_districts_shaded_(2016).svg; Htonl, CC BY-SA 3.0, via Wikimedia Commons).

The study area was the whole of the ECP (Figure 1), and the study formed part of a larger study that also looked at poultry diseases [12]. The province has a population of 6,676,590 people [13] and has one of the highest unemployment rates in the country [14]. The informal pig sector in the ECP is estimated to have 536,108 pigs [15], most of which are found in the 6024 villages disseminated in the province [16].

#### 2.1.3. Sampling Procedure

The methodology included a chicken survey conducted in the province; hence, the sampling procedure and questionnaire interviews used the same study design described and published in a parallel study [12]. The calculated number of households to be surveyed in the study was 495, which was rounded to 500 households. This was divided into 250 chicken farmers and 250 pig farmers [12]. The questionnaire contained sections on farm owner demographics (gender, age and level of education), farming practices, (e.g., use of swill and contact with African wild suids), trading practices and biosecurity measures. Questions related to pig diseases and the remedies used to treat them over the past 12 months were also included in the questionnaire. The term “remedy” is used in the context of a medicine, application or treatment that relieves or cures a disease1 and thus includes any traditional and conventional medicinal substance. For biosecurity measures, farmers were asked if they had measures in place to prevent or control diseases on their farms. They were thereafter asked to give details about the nature of these measures if the response was “yes”. A list of biosecurity measures applicable to smallholder pig farms has been described in more detail in a related publication [4]. Information on trading practices and value chain for the farmers in this study are dealt with in a separate paper [17].

#### 2.1.4. Data Analysis

All data from the questionnaire were analysed using the software programmes Epi Info® 7, NCSS® and Microsoft Excel for descriptive statistics. Fisher's exact test was used to determine the statistical difference between the number of males and females interviewed [18]. A more detailed explanation of statistical methods has been published in a related study [12].

## 3. Results

### 3.1. Demographics of Smallholder Pig Farmers Interviewed

Among 214 smallholder farmers who were interviewed, 111 were females (52%) and 103 were males (48%), (*p*=0.44) confirming that a large proportion of farmers were females. For analysis, farmers who were interviewed were grouped into three categories according to their age: young adults (from 0 to 35 years), adults (36–55 years) and pensioners (56–89 years). The survey showed that pensioners were more represented (52.3%), followed by adults (36%) and young adults (11.7%). The majority of smallholder pig farmers (40.7%) had primary education (from grade 1–9), followed by farmers with secondary education (grade 10–12) (35%). About 14.5% of smallholder pig farmers had tertiary education, whereas 9.8% of farmers had no formal education (Table 1).

### 3.2. Farming Practices Related to Poor Biosecurity in the Province

#### 3.2.1. Farming System and Use of Swill

The questionnaire survey revealed three types of feed used by pig smallholder farmers: commercial feed, supplements (crushed maize) and kitchen waste (swill). The majority of farmers (72.4%) confined pigs in one area and fed them commercial feed with regular supplementation (intensive system), while 17.8% housed their pigs and fed them commercial feed with occasional supplementation but allowed them to move around the farm to scavenge within an enclosed area within the farm area (semi-intensive system) and 8.4% allowed their pigs to scavenge around the village or beyond with no proper housing, feed or supplementation (free range system) (Table 1). Some smallholder pig farmers (1.4%) did not specify how their pigs were managed. A large portion (75.7%) of smallholder pig farmers used kitchen waste (swill) in addition to the commercial feed and supplements (Table 1).

#### 3.2.2. Contact of Domestic Pigs With African Wild Suids

A number of farmers (5.6%) reported their pigs were sharing a common habitat with African wild suids (Table 1).

#### 3.2.3. Trading Practices

Only a small percentage of farmers (15.9%) traded pigs on a regular basis (every 6 months or less), while many farmers (48.1%) did not regularly sell pigs (at least once a year). Farmers who were not involved at all in trade represented 35.9%. The percentage of farmers selling pigs through auctions was 0.9%. None of the farmers (0%) obtained a movement permit or a health certificate from veterinary services before trade (Table 1).

#### 3.2.4. On-Farm Biosecurity and Disease Prevention Practices

All the farmers who were interviewed had poor biosecurity measures in place to prevent pig diseases coming into the farm. Many farmers reportedly used a mixture of remedies to treat any signs of disease in pigs. Remedies used by smallholder pig farmers to treat or prevent pig diseases were subdivided into six categories: traditional, antibiotic, antiparasitic, acaricide, anthelmintic and vitamins and minerals. The most representative category of remedies was antibiotics, used by 31.1% of farmers, followed by traditional remedies, used by 18.5% of farmers. Farmers who used antiparasitic drugs represented 15.6% of farmers, whereas those who used vitamins and minerals, acaricide and anthelmintics represented 6.6%, 4% and 2.3% of the farmers, respectively. Farmers who did not report the use of any remedies to treat pig diseases made up 21.9% of the farmers (Table 2).

## 4. Discussion

The number of female smallholder pig farmers was slightly higher (52%) than the number of males (48%). Although the difference between males and females was not statistically significant, the representation of female smallholder pig farmers reflects the cultural importance of this subsector in Xhosa culture, the most predominant in the province. Women in rural communities have an obligation to be involved in pig and poultry husbandry, while men manage other species (Batyi, unpublished data). Similar findings were noted in the rural pig and poultry sector of ECP where female smallholder farmers were more represented than males [5, 10, 12, 19], highlighting their socioeconomic importance in providing the basic household needs (i.e., food and school fees) [20]. When analysing the age-group category, pensioners were more represented compared to young adults and adults, highlighting the importance of pigs as an additional income generating activity for this segment of the community. This is important for food security considering the inadequate welfare programmes in many African countries. A similar finding was noted in a recent survey of village chicken farmers in the province where pensioners were more represented in poultry farming than any other age category [12]. Given the high unemployment rate in the province [21], the expansion of the smallholder pig industry could contribute to job creation and become a source of income for adults having difficulty finding permanent employment.

Smallholder pig farmers with only a primary school level of education made up the highest proportion of pig farmers (40.7%) compared to those with secondary and tertiary education level. Similar findings were reported in other studies of the primary industry in the ECP where farmers with a low level of education were more represented [12, 19, 22]. This could explain why farming in the ECP is still traditional and underdeveloped despite the high number of livestock in the province [22]. The level of farmers' education is known to influence their scope of decision-making, and this is related to the success of a farming business [23].

The low level of education could also possibly explain the low biosecurity measures in this informal pig sector because most pig farmers who were interviewed seemed not to be aware of the importance of biosecurity in preventing pig diseases. Instead, they were relying on remedies to treat and prevent pig diseases. This finding was supported by similar studies carried out elsewhere, where farmers relied on the use of remedies instead of applying basic biosecurity measures to prevent pig diseases [24–26]. In this study, the most representative category of remedies used by smallholder pig farmers was antibiotics (31.1%), with tetracyclines and sulpha products being the most used remedies (Table 2). Tetracyclines were also reported to be the most used antibiotic in smallholder pig farming in Limpopo Province [27]. The availability and use of these antibiotics by smallholder pig farmers coupled with a lack of knowledge and training on antibiotic use could contribute to antimicrobial resistance (AMR), which has become a public health concern in the last decades. The present study found that farmers had access to these antibiotics as over-the-counter medicines through local private livestock pharmaceutical suppliers. Antimicrobial use in both human and animals has been responsible for the emergence and spread of AMR in bacterial populations, resulting in increasing antimicrobial therapy failure [25]. Many farmers did not report using any remedies to prevent or treat sick pigs (21.9%), which is probably a reflection of their socioeconomic status. Traditional remedies also occupied an important place among remedies used by smallholder pig farmers (18.5%). A similar finding was noted in a study of village chickens where many farmers relied on traditional remedies to prevent and treat chicken diseases [12], with *Aloe ferox* Mill. *(Asphodelaceae)* or “ikhala” (in local language) being used in both chickens and pigs. Another frequently used remedy identified was macrocyclic lactones (antiparasitic), which was mainly used to treat skin disease (mange). This group of remedies was found to be cost-effective in pigs in another study, since it could be used for both external and internal parasitic infestations [28].

The analysis of pig farming systems revealed that a free-ranging system was practiced by 8.4% of pig smallholder farmers, which represents a risk for rapid disease transmission and spread when there is an outbreak. Although the majority of pig keepers interviewed (72.4%) reported the use of intensive production systems, it was found that many pigs were kept in very poor housing structures from where they could easily move in and out and wander around the village. The lack of proper pig housing structures, therefore, makes the implementation of biosecurity for smallholder pig farmers difficult in the province. Similar poor housing structures of pigs were also found in a study in Limpopo Province [27]. In areas where a cycle between pigs and tampans (*Ornithodoros* sp.) exists, housing pigs in structures that offer a suitable habitat for the ticks also poses a risk for the emergence of ASF [1].

Informal trade has been mentioned in previous studies as a major risk factor for ASF transmission in domestic pigs [2, 29–31]. Our study found that some backyard pig producers in the province were reportedly selling live pigs and pig products across the province without meat inspection or a health permit (informal market), thus contributing to the risk of disease spreading from smallholder farms. A segment of this informal market was reported to be more profitable than the formal one. In the formal market, pigs were sent to an abattoir for slaughter and meat inspection, but with less incentive given to the farmer [17]. Consequently, many farmers who were interviewed reported that slaughtering and selling pork without using abattoirs (informal market) was more profitable for them [17]. The lack of transport and inaccessibility to the market by smallholder pig farmers in the ECP have been also mentioned as a challenge [9]. The lack of proper meat inspection with informal slaughtering, and consequent failure to detect diseases that may be present, could contribute to the transmission and maintenance of diseases in local pig populations [3]. This practice was also found to contribute to the propagation of *Taenia solium* cysticercosis, the causative agent of neurocysticercosis in the rural community of ECP [9]. The prevalence of important neglected diseases, such as leptospirosis, in rural communities of ECP remains largely unknown [32]. A study on trading practices of pig farmers and movement of live pigs and their products in the ECP would give more insight into the epidemiology of pig diseases in the region.

A high number of smallholder pig farmers (75.7%) used untreated kitchen waste (swill) when feeding their pigs. Feeding of swill containing pig remains has shown to play a role in the transmission of ASF in domestic pigs [33, 34]. The practice of swill feeding could be due to the lack of knowledge of the risks involved but is probably because these smallholder farmers could not afford using commercial feed alone. This finding was also reported in the Northern Cape and Free State Provinces where the practice of swill feeding was more likely due to the cost implications of obtaining commercial feed, especially when the costs in obtaining feed would most probably make the enterprise unprofitable within the available marketing options [3]. Farmers who were interviewed reported not using meat as part of swill, but this information could not be verified. In addition, untreated kitchen or restaurant waste could contain meat products without a farmer's knowledge [3]. These risky practices could be reduced or eliminated by developing simple and cost-effective biosecurity measures and marketing opportunities that provide an incentive for investment and modernisation of the pig industry [2, 35]. A participatory approach to promoting biosecurity is recommended, as it ensures disease prevention or control actions are guided by local people's priorities and it promotes local ownership of disease control [36].

## 5. Conclusion

This is the first study describing the sociodemographics of smallholder pig farmers in the ECP and their practices related to the spread of communicable pig diseases in the province. Farming systems that involve free-range pigs, swill feeding and informal trade were identified as practices that could contribute to the spread of diseases in the province. A low level of education and reliance on remedies to treat and prevent pig diseases could explain the poor biosecurity measures on their farms. Smallholder pig farming in the province, therefore, increases the risk of incursion and spread of pig diseases, posing a risk for commercial farms. A lack of knowledge and training on the use of antibiotics could be contributing to AMR in rural pigs. There is, therefore, a need to train smallholder pig farmers in biosecurity and antibiotic usage to improve disease control and prevent AMR. Providing marketing opportunities would provide an incentive for investment and modernisation of the rural pig industry.

## 6. Limitations of the Study

It was not always possible to get 15 smallholder pig farmers per local municipality on the day of interviews; hence, the obtained number of 214 smallholder farmers who were interviewed instead of 250 farmers who were targeted in the study design.

## Figures and Tables

**Table 1:** Demographics and farming practices identified during the survey in the Eastern Cape Province (February–June 2019).

	Percentage of respondents
Demographics	
Gender	
Females	52% (111/214)
Males	48% (103/214)
Age	
Young adults (0–35)	11.7% (25/214)
Adults (36–55)	36% (77/214)
Pensioners (56–89)	52.3% (112/214)
Level of education	
None	9.8% (21/214)
Primary (Grades 1–9)	40.7% (87/214)
Secondary (Grades 10–12)	35% (75/214)
Tertiary	14.5% (31/214)
Farming practices	
Farming systems	
Intensive	72.4% (155/214)
Semi-intensive	17.8% (38/214)
Free range	8.4% (18/214)
Not specified	1.4% (3/214)
Feeding of swill	75.7% (162/214)
Contact with African wild suids	5.6% (12/214)
Selling pigs through auctions	0.9% (2/214)
Trading activity on a regular basis (every 6 months or less)	15.9% (34/214)
Trading activity at least once a year	48.1% (103/214)
Farmers not involved in trade	35.9% (77/214)
Movement permit or health certificate before trade	0% (0/214)

**Table 2:** Remedies used by smallholder pig farmers in the Eastern Cape Province according to the survey conducted between February and June 2019.

Category	Remedies	Active ingredient	Frequency of usage (%)
Not using any remedy^∗^	—	—	

*Antibiotics* ^∗∗^ *:*			
Tetracyclines	Terramycin, Hi-Tet	Oxytetracycline HCl	18.2%
Sulpha products	Norotrim	Sulphonamide	11.9%
	Sulfazine	Sulphadimidine sodium	16%
Penicillin	Duplocillin	Procaine benzylpenicillin	1%

Traditional	Sibabile	Unknown	18.5%
	Zifozonke	Sodium permanganate	
	Madubula	Tar acid	
	Ashes	Unknown	
	Salt	Sodium chloride	
	Sunlight soap	Unknown	
	Engine oil	Unknown	
	Epsom salts	Magnesium sulphate	
	*Aloe ferox* Mill.	Cape *Aloe ferox* gel, vitamins C, B5, A, E, B6 and B2	
	Sugar		

Antiparasitic macrocyclic lactones	Dectomax, ivermax	Ivermectin	15.6%

Vitamins and minerals	Multivite, calcium, iron dextran	Vitamins A, B, C, calcium, iron hydrogenated dextran	6.6%

Acaricide	Dazzel N.F.	Diazinon 30% m/v	4%

Anthelmintic	Piperazine salts	Piperazine citrate	2.3%

^∗^Farmers who were not using any remedy to treat pig diseases represented 21.9%.

^∗∗^Combined antibiotic use (tetracyclines, sulpha products and penicillin): 31.1%.

## Data Availability

The data that support the findings of this study are available from the corresponding author upon reasonable request.

## Endnotes

^1^ https://www.merriam-webster.com/dictionary/remedy. Accessed 7 Apr. 2025.
